# Co‐Design and Feasibility Testing of an AI‐Based Virtual Reality Application to Prepare People With Intellectual Disability for Healthcare Visits

**DOI:** 10.1111/jar.70219

**Published:** 2026-04-08

**Authors:** Stefan C. Michalski, Jane Adams, Ali Darejeh, Rachael C. Cvejic, Sylvia M. Gustin, Julian N. Trollor

**Affiliations:** ^1^ National Centre of Excellence in Intellectual Disability Health, Faculty of Medicine and Health UNSW Sydney Sydney New South Wales Australia; ^2^ School of Computer Science and Engineering, Faculty of Engineering UNSW Sydney Sydney New South Wales Australia; ^3^ NeuroRecovery Research Hub, School of Psychology, Faculty of Science UNSW Sydney Sydney New South Wales Australia

**Keywords:** artificial intelligence, healthcare access, intellectual disability, qualitative, virtual reality

## Abstract

**Background:**

People with intellectual disability experience barriers in accessing healthcare. A virtual reality (VR) application that includes an intelligent agent powered by generative artificial intelligence (AI) may support preparation for healthcare visits in this population.

**Objective:**

To co‐design, develop and evaluate the feasibility and usability of an AI‐based VR application to improve healthcare preparedness for people with intellectual disability.

**Methods:**

Ten adults with intellectual disability completed an AI‐VR experience simulating a general practitioner visit across three sequential scenes: checking in with a receptionist, waiting in a clinic waiting room, and consulting with a doctor. Participants interacted verbally with AI avatars. Semi‐structured interviews followed each scene.

**Results:**

Participants valued the avatars' clear, patient communication and described the system as supportive for learning healthcare content and practising communication and self‐advocacy skills. Usability issues were identified.

**Conclusion:**

AI‐VR appears feasible and acceptable for healthcare preparation in people with intellectual disability. Refinements to system usability are needed to support independent use and broader implementation.

## Lay Summary

- We developed a virtual reality programme using artificial intelligence to help people with intellectual disability prepare for healthcare visits.
- Ten people with intellectual disability tested the programme. They liked the clear communication and found it helpful for learning and practising questions.
- Speech recognition errors and other technical problems meant people needed some help to use the programme.
- The study shows what is needed to make virtual reality programmes accessible for people with intellectual disability.

## 1 Introduction

People with intellectual disability experience severe health disparities, including higher rates of preventable hospitalisations (Weise et al. 2021), higher rates of potentially avoidable deaths, and death earlier than the general population (Trollor et al. 2017). Despite an elevated risk profile, people with intellectual disability have higher unmet health needs (Bauer et al. 2019; Byrne et al. 2016; Salvador‐Carulla and Symonds 2016) and lower uptake of preventative health services such as screenings, regular check‐ups, and vaccinations (Emerson et al. 2011; Felce et al. 2008; Havercamp and Scott 2015). People with intellectual disability experience many barriers accessing healthcare that have been identified decades ago (Lennox et al. 1997), and remain unaddressed today (Doherty et al. 2020; Shea et al. 2022; Barrington et al. 2025). The Australian healthcare system has failed to meet the needs of people with intellectual disability (Trollor 2020). Innovative approaches to improve healthcare access for this population are therefore needed.

Healthcare access barriers operate at both the systemic and individual level for people with intellectual disability. Systemic barriers include limited accessible information (Geukes et al. 2018; Ali et al. 2013; Powrie 2003), time constraints within healthcare appointments (Burton and Walters 2013; Brown et al. 2017; Zarotti et al. 2022), lack of knowledge and training of health professionals in intellectual disability health (Rinaldi and Batsele 2023; Northway et al. 2017; Cross et al. 2012; Newton and McGillivray 2019; Navas et al. 2019), and stigma or discrimination (Donner et al. 2010; Tuffrey‐Wijne et al. 2014; Agaronnik et al. 2020; Jones et al. 2008). At the individual level, anxiety (Lennox et al. 1997; Burton and Walters 2013), fear and embarrassment (Codling 2015; Miller et al. 2008; Raymaker et al. 2017), and a general lack of trust due to past negative experiences (Donner et al. 2010; Cox et al. 2021; Gibbs et al. 2008; Weiss and Lunsky 2010), are commonly reported barriers (Doherty et al. 2020; Shea et al. 2022). Whilst systemic barriers require broad structural changes to healthcare delivery, individual barriers related to anxiety, communication skills, and healthcare preparedness present opportunities for targeted interventions that can directly address modifiable factors affecting healthcare access.

The National Roadmap for Improving the Health of People with Intellectual Disability in Australia recognised these access challenges and included a strategic action to explore the use of emerging technologies to reduce barriers to health care (Australian Government Department of Health 2021). Virtual reality (VR) is gaining interest amongst researchers and disability support professionals for its potential to enable experiential learning opportunities. People with intellectual disability often experience difficulties processing abstract concepts and generalising skills across contexts (Spaniol and Danielsson 2022; Hronis et al. 2017). VR enables learning through direct experience in controlled and repeatable simulations that break down complex real‐world experiences into manageable steps. Users can practise skills and build confidence in unfamiliar settings without relying on verbal instruction or abstract reasoning. Importantly, there is growing evidence demonstrating that immersive VR is both well tolerated and effective for supporting skill development in people with intellectual disability, including training life skills (Franze et al. 2024; Michalski et al. 2023; Nabors et al. 2020). The application of VR in healthcare for people with intellectual disability, however, remains limited.

Most existing VR health research for people with intellectual disability focuses on physical fitness interventions and motivation to engage in exercise activities (Li et al. 2023; Mocco et al. 2024) and distraction‐based interventions during medical procedures (Mehrotra et al. 2023; Mehrotra et al. 2024). Some studies have aimed to prepare people for healthcare interactions, used computer‐based programmes for autistic individuals (Boada and Parellada 2017) and people with intellectual disability (Hall et al. 2011), but these were neither immersive nor interactive. More recently, Acton et al. (2024) co‐designed and developed an immersive VR experience that incorporated personalised videos of healthcare staff, with clinicians providing brief introductions and sharing information about themselves. The VR experience aimed to build rapport by helping participants become familiar with staff prior to appointments. The study found statistically significant reductions in fear scores and improvements in quality of life. Building on this foundation, there is potential to enhance VR environments with interactive capabilities that enable users to practise communication and engage in real‐time exchanges with healthcare staff.

Integrating artificial intelligence (AI) into VR offers an opportunity to provide personalised healthcare preparation for people with intellectual disability. AI‐based conversational agents can answer users' specific questions or concerns before attending an appointment. In the immersive VR environment, users can practise common healthcare activities including checking in for appointments, experience the waiting room environment, and practise asking questions to the doctor. Early work has demonstrated the potential for AI‐based conversational agents in related contexts, with applications such as job interview training for young adults with autism spectrum disorder showing promising outcomes in augmented reality environments (Hartholt et al. 2019). AI‐based VR experiences can provide visual cues and step‐by‐step guidance that accommodate individual communication styles and learning needs, potentially reducing anxiety, increasing comfort, and improving willingness to seek care.

The primary goal of this study is to co‐design, develop, and evaluate the feasibility and usability of immersive AI‐based VR applications to improve healthcare preparedness for people with intellectual disability. Specifically, the study aims to: (1) Co‐design and develop an AI‐based VR application simulating typical healthcare environments and interactions, such as attending a general practitioner's (GP's) clinic; (2) Evaluate the feasibility and usability of the application through user testing with people with intellectual disability; (3) Identify factors that influence engagement with AI‐based VR to make people feel more prepared for healthcare interactions.

## 2 Materials and Methods

### 2.1 Co‐Design

The co‐design process involved two sequential 2‐h meetings held 5 months apart, including people with intellectual disability and a facilitator from the *Council for Intellectual Disability* (NSW, Australia). The first session focused on barriers to healthcare for people with intellectual disability and explored how VR might address these challenges. Participants described difficulties with communication, lack of consent, feeling rushed, and sensory stressors such as loud noises and bright lights. They emphasised the need for clear explanations, empathy from healthcare providers, and being informed about what to expect. During this co‐design session, participants tried an off‐the‐shelf VR experience to get an understanding of VR. Participants identified top priorities for a VR healthcare preparation tool: practise talking with doctors, see what the clinic looks like beforehand, reminders of what to bring to an appointment, and use VR in a safe space with support. Participants and facilitators discussed preparation for general practitioner (GP) visits. Primary care is the main entry point to the healthcare system and the setting where preventative care and early management of health concerns occur. The group therefore identified GP appointments as an appropriate starting point for developing a healthcare preparation tool. Participants stressed the importance of clear, respectful communication and preferred easy‐to‐read adult information that respected their autonomy. Following this session, a VR prototype was developed based on the top priorities identified by the group. In the second session, participants reviewed screen recordings of the VR prototype and provided feedback on the reception area, waiting room, and doctor's office scenes. Key insights included making conversations feel more natural and realistic, ensuring environments look like real GP settings, including common reception steps like checking in and payment, and adding clear examples of what to expect during visits. These refinements were implemented for the next iteration to be tested in the research study.

### 2.2 Study Design

This study evaluated the feasibility and usability of an AI‐based VR application through participant testing and feedback. Semi‐structured, open‐ended questions were used to capture participants' reflections following the AI‐VR experience. Interviews and AI‐generated transcripts were audio‐recorded, transcribed, and analysed thematically.

### 2.3 Participants

Ten participants with intellectual disability completed the research study. Participants were recruited from a disability advocacy organisation (NSW, Australia) that we have partnered with in multiple previous research and capacity building projects. As members or active contributors to the organisation, participants had experience engaging in peer‐led initiatives and were familiar with giving feedback on programmes aimed at improving accessibility and inclusion.

Inclusion criteria for participation required that participants be adults aged 18 years or older with intellectual disability who could speak English, ability to wear a virtual reality headset, and hold a handheld controller, and were not involved in the co‐design of the application. These criteria were selected to support safe use of the VR equipment and to enable direct verbal interaction with the AI conversational system, which relied on speech input in the current prototype. Exclusion criteria included people who did not meet the inclusion requirements, people who were involved in co‐design of the application, and people with epilepsy, as recommended by Birckhead et al. (2019). The concept of ‘information power’ (Malterud et al. 2016) was applied to determine whether sufficient data had been obtained. Table 1 presents characteristics and baseline healthcare experiences of the study participants.

**TABLE 1:** Participant characteristics and baseline healthcare experiences.

	Responses
Demographic information	
Age in years (mean, range)	42.9 (27–67)
Gender	Female: 5
	Male: 5
At least one co‐occurring health or mental health condition	5 (50%)
General information about healthcare access	
Who usually goes with you to doctor's appointments?^a^	Family/Partner: 5
	Support worker: 6
	Alone: 2
How do you feel when you go to the doctors?^a^	Happy/Comfortable: 5
	Anxious/Nervous: 4
	Worried/Scared: 4
	Mixed: 3
Do you understand what the doctor tells you?	Yes, always: 2
	Sometimes: 6
	No, not often: 1
	N/A: 1
Do you feel comfortable asking the doctor questions?	Yes, always: 6
	Sometimes: 4
	No, not often: 0
Do you feel the doctor listens to you?	Yes, always: 4
	Sometimes: 4
	No, not often: 2
Patient reported experience from last healthcare visit	
Did the people who care for you at the clinic find out how you like to communicate?	Yes: 6
	Sometimes: 4
	No: 0
Did the people who work at the clinic listen to you?	Yes: 7
	Sometimes: 2
	No: 1
Did the people at the clinic give you clear instructions?	Yes: 7
	Sometimes: 2
	No: 1
Did the people who work at the clinic tell you things in a way that you understand?	Yes: 8
	Sometimes: 1
	Not really: 1
Did the people at the clinic let you ask questions?	Yes: 7
	Sometimes: 1
	No: 2
Did you feel safe at the clinic?	Yes: 6
	Sometimes: 3
	No: 1

^a^ Participants could select multiple responses.

### 2.4 Materials

#### 2.4.1 VR Apparatus

The Meta Quest 3 head‐mounted display (HMD) was used. Whilst wearing the HMD, participants viewed a three‐dimensional environment that moved in accordance with their movements in real time. Participants moved freely in the physical environment while their movement was reflected in the virtual environment. Eyeglasses and hearing aids could be worn under the device if needed.

The device includes handheld controllers that allow users to interact with and navigate the virtual environment. The researcher held the controller throughout the session to guide the virtual experience. The researcher remained within arm's reach of the participant to assist if needed.

#### 2.4.2 AI‐Based VR Application

**FIGURE 1:**
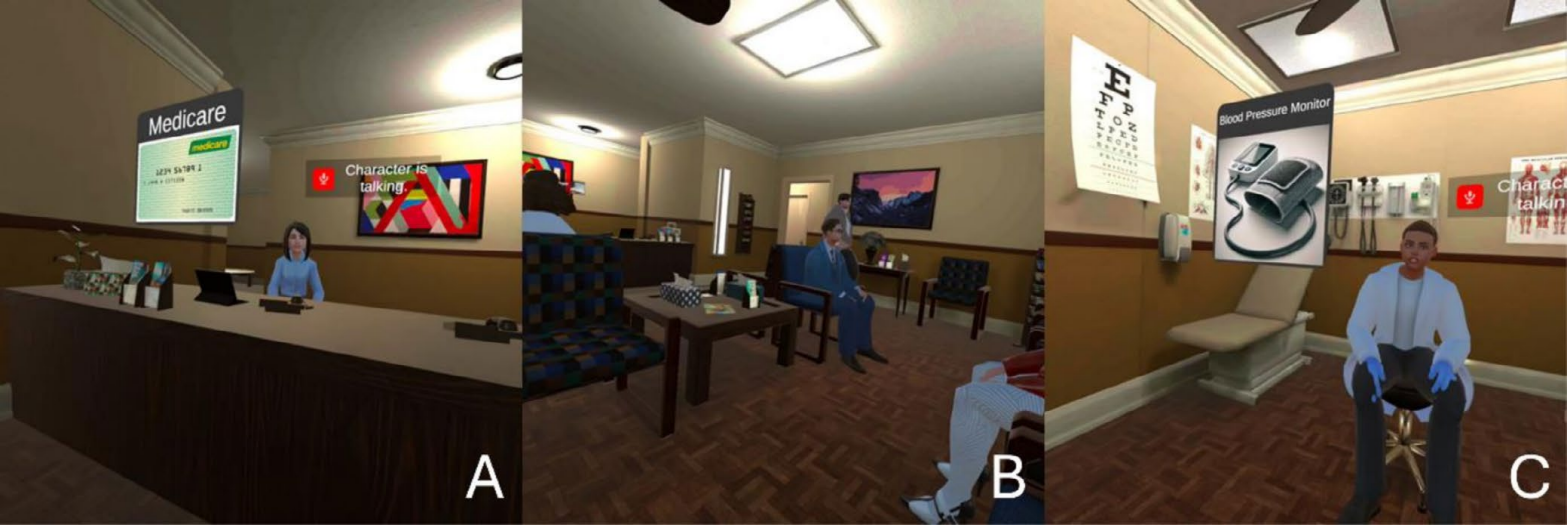
Virtual reality environment. Scene 1—Checking in with the receptionist (A). Scene 2—Sitting in the waiting area (B). Scene 3—Speaking with the doctor (C).

The co‐designed AI‐based VR application simulated a GP visit through three sequential scenarios: checking in with a receptionist, waiting in a clinic waiting room, and consulting with a doctor (see Figure 1).

*Scene 1: Check‐in with the Receptionist*. Participants approached an AI‐receptionist who welcomed them to the virtual experience. The receptionist presented five items that you should bring to your healthcare appointments (including a health insurance card and concession card). Each item appeared in the simulation and the receptionist explained its purpose. Participants could ask questions about each item as it was introduced. This scene took approximately 5–10 min.

*Scene 2: Waiting Room Simulation*. The second scene depicted a clinic waiting room with several avatars, background noise from a television, and ambient sounds such as phone calls and people coughing or sneezing. Some avatars sat quietly whilst others moved around or spoke to one another. Participants remained seated and waited until the doctor avatar entered the room (approximately 2 min).

*Scene 3: Doctor's Consultation*. Participants interacted with an AI‐doctor. The doctor introduced themselves, explained the privacy and safety of the consultation space, and encouraged participants to examine the room. The doctor presented a series of common medical instruments (e.g., stethoscope and reflex hammer) with simple explanations of each instrument's function. Participants could ask questions about each instrument as it was introduced. If participants asked about the treatment of a specific medical condition, the AI‐doctor redirected them to seek advice from a real healthcare provider. This scene took approximately 5–10 min.

The AI‐VR application was developed using Unreal Engine 5. Development was conducted on high‐performance workstations equipped with Intel Core i9 processors, NVIDIA GeForce RTX graphics cards, and 32 GB RAM. The environment was modelled using a combination of Unreal Engine assets and custom 3D models created in Blender. AI conversational functionality was implemented through integration of the OpenAI ChatGPT‐4 API, accessed via Convai middleware (www.convai.com). This allowed real‐time communication between the participant's spoken input (captured through the Meta Quest 3 microphone) and AI‐driven avatars. Scene logic, avatar behaviour, and interaction flow were programmed using Unreal's Blueprint Visual Scripting System. The final build was deployed to the Meta Quest 3 as a standalone executable using Unreal's Android‐based build pipeline.

### 2.5 Measures

#### 2.5.1 Demographic and Healthcare Information

A demographic questionnaire collected information including age, gender, and self‐identification as a person with intellectual disability. The questionnaire assessed healthcare access patterns, emotional responses to healthcare visits, communication barriers, and comfort during appointments. Select items from an early version of the *Listen to Me Patient‐Reported Experience Measure (PREM)* were also used to assess patient experiences at participants' most recent healthcare visit. The *Listen to Me PREM* is a co‐created patient‐reported experience measure developed for people with intellectual disability (Harrison et al. 2025).

#### 2.5.2 Semi‐Structured Interviews

Following each VR task, semi‐structured interviews were conducted to collect qualitative feedback about participants' experiences. For each VR scene, participants described their experience, identified what they liked and disliked, and compared the virtual environment to their real healthcare experiences. Participants were asked whether they found interacting with virtual characters difficult, how these characters compared with real healthcare staff, and what other situations could be included in the programme. At the end of the VR experience, participants identified their preferred aspects and suggested improvements. Additional questions explored emotional responses, comfort, and whether the VR experience could support real‐life healthcare visits. The questioning framework provided structure whilst allowing flexibility to ask follow‐up questions, request clarification, and adjust pace and language to support individual participants.

#### 2.5.3 Task Performance Data

The conversational AI software platform automatically captured and transcribed all verbal interactions between participants and AI conversational agents during the VR experience. These transcripts were checked against audio recordings to ensure accuracy and identify instances where the AI agent had misunderstood participants. Key metrics included the total number of verbal communicative acts (yes/no responses, questions, and comments), the proportion of valid AI responses, microphone activation issues, and technical issues. Valid responses were defined as AI responses that appropriately addressed user input, including moving to the next scripted segment after a yes/no response, answering a question, or responding on topic to a comment. Microphone status was monitored to identify instances where participants spoke before the microphone was activated (indicated by a green microphone signal).

### 2.6 Procedure

Data collection was completed at the UNSW Sydney Kensington campus. Upon arrival, participants provided written consent and completed the baseline questionnaires. The researcher introduced the VR equipment and explained the session activities, including the instruction to wait for a green microphone signal before asking questions to the AI avatars. Participants were told that they could ask the AI avatars questions or make comments about the topics being discussed. The Meta Quest 3 headset was fitted to each participant. Participants were initially required to be able to operate the VR hand controller independently as part of the inclusion criteria. However, during early data collection, it became apparent that controller operation posed a barrier to engagement. The protocol was modified so that the researcher operated the hand controller for all participants, allowing them to focus on verbal interaction with the AI avatars. Following each VR scene, the researcher conducted semi‐structured interviews to gather qualitative feedback. All verbal responses during the VR experience and interviews were audio‐recorded and transcribed for analysis. The researcher took field notes to document technical issues, participant reactions, and contextual observations.

### 2.7 Qualitative Analysis

A reflexive thematic analysis approach was used (Braun and Clarke 2006; Braun et al. 2023). JA conducted initial coding of all transcripts to establish preliminary coding categories. SCM then reviewed the transcripts and codes, and both researchers collaboratively identified relationships amongst recurring and conceptually significant codes to construct thematic frameworks. JA maintained detailed notes throughout this process to record decision‐making rationales and theme development progression. The research team convened regularly to discuss and refine proposed themes. When disagreements arose, these were resolved through discussion until consensus was reached, and the process of refining themes continued during the drafting of the manuscript. NVivo 14 was used for data management.

### 2.8 Position of the Researchers

SCM is a Research Fellow at the National Centre of Excellence in Intellectual Disability Health (National Centre) at UNSW Sydney who completed his PhD examining the use of immersive virtual reality to build capacity in people with intellectual disability. JA is a PhD candidate in Social Sciences at UNSW Sydney with doctoral research focusing on disability and NDIS experiences. She has over 10 years of clinical experience as a speech pathologist working with children and young people with disability and their families and holds a master's degree in Developmental Disability. Both researchers have experience researching healthcare barriers affecting people with intellectual disability.

Based on co‐design session feedback, both researchers anticipated overall positive participant responses to VR technology whilst expecting varied individual reactions based on participants' diverse healthcare experiences and technology familiarity. Neither researcher had prior contact with study participants before recruitment. Both researchers' institutional affiliation with the National Centre may have influenced participant expectations. JA conducted all interviews and led initial coding. To address potential bias, JA maintained reflective documentation throughout data collection and analysis phases, with theme development involving collaborative discussion until consensus was reached. No external validation methods such as member checking or independent coding verification were employed.

## 3 Results

### 3.1 Task Performance

Quantitative data were extracted from AI‐generated transcripts to examine participant engagement, communication patterns, and system responsiveness during the VR task. Table 2 summarises the key task metrics for the sample.

**TABLE 2:** Task metrics recorded during the AI‐VR experience.

Metric	Average (range)	Percentage of valid responses by AI
All communicative acts	13.3 (6–37)	88.3%
Questions asked by the user	3.7 (0–28)	91.9%
Comments made by the user	1.6 (0–10)	53.1%
Attempts to speak when the microphone was inactive	2.1 (0–8)	—
Technical issues	3.1 (0–9)	—

*Note:* All communicative acts include yes/no responses + questions + comments. Technical issues refer to when the researcher needed to intervene for reasons such as needing to prompt to rephrase the question or assist with interpreting the AI response.

### 3.2 Thematic Analysis

Thematic analysis of interview data and participant‐AI interaction transcripts identified three main themes (Table 3). Themes were identified inductively through iterative coding and analysis of the data. For Theme 2, concepts of presence and immersion were incorporated after initial coding using existing theoretical frameworks (Slater 2018; Wilkinson et al. 2021; Witmer and Singer 1998). All ten participants contributed experiences across these themes.

**TABLE 3:** Summary of themes from the thematic analysis.

Theme	Description
Theme 1: A Human Touch in a Virtual Conversation	Learning healthcare content through personalised conversations with AI avatars that functioned as patient, supportive learning companions
Theme 2: Stepping Into the Virtual Clinic	Experiences of immersion in virtual healthcare environments, with environmental realism and avatar behaviour affecting engagement and healthcare preparation
Theme 3: The Promise and Problem of VR	Recognition of VR educational potential alongside technical barriers (microphone activation, speech recognition) that limited independent use

#### 3.2.1 Theme 1: A Human Touch in a Virtual Conversation

This theme captures participants' experiences of learning healthcare content through personalised conversations with AI avatars. The AI system functioned as a learning companion that provided an overview of what to expect in a healthcare clinic and answered specific questions users had about the content provided. The experience enabled participants to engage through interactive dialogue, providing an opportunity for active questioning and self‐advocacy skills. Most participants described some positive aspects of the AI communication, with participants distinguishing between their experiences with the ‘receptionist’ and ‘doctor’ avatars.

Participants consistently valued the AI avatars' clear communication style and explanations that were easy to understand. Participants were mostly positive about the accessible language used by the AI receptionist and doctor, with Participant 9 noting that: ‘… I can listen much easier because it's plain English. It's not hard for me and she speak very, very clear.’ All participants initially received consistent scripted information about healthcare appointments. When participants asked follow‐up questions, the AI system provided unscripted responses that were longer and more complex than the original scripted content. Some participants noted that responses to their questions required simpler language, as noted by Participant 8: ‘Just need to like speak, maybe in a simple way, as like the doctors, the same thing too. Maybe simplifying a bit more, but yes it did explain it.’ (Participant 8).

Throughout their interactions, participants experienced the AI system as more than just an information provider as it functioned as a supportive learning companion that guided them through healthcare scenarios at their own pace. The AI system provided supportive communication that helped participants feel comfortable, reflecting the relational nature of some real‐life healthcare interactions.

…seeing him of explaining to me about everything, that's already make me feel comfortable, because I can even see his face. He's calm. He's not rushing me or anything. He's very patient with me. He is answering me my questions. … He's waiting for me to think about my questions. He doesn't really tell me. He doesn't cut me off when I'm still talking or anything. (Participant 10)

The content of the VR programme was mostly considered less emotive than real life healthcare appointments, where participants were sometimes worried about the results of tests, or worried about a healthcare issue. Some participants described how emotional aspects of real healthcare appointments impacted on their ability to understand new information, whereas the VR experience allowed more time to settle into the experience and feel receptive to the information provided.

I felt, like, [the VR] does help a lot … that was mostly make you feel calm a lot of time [more] than going to your normal doctor … mostly us [people with] disability, we don't focus [on] what doctors say. We first know what is happening around us and we first look what he the doctor's carrying, you know and when it's carrying a needle or anything, start freaking out. … I was like, looking at the [VR] office, and I saw that, OK, there's nothing that's [going to] harm me or anything and I feel comfortable after that. (Participant 10)

Participants perceived the avatars as being patient and unrushed, a characteristic that was particularly valued by those who needed time to process responses and formulate thoughts. The AI system often had a short delay whilst processing a response to participants' questions, which served well to slow down the pace of the conversation and contributed to the perception of the avatars as patient.

I like when the [real life] doctor like speak to you, like easy words and explain things. And listen, listen, listen, listen and understand what I'm saying too. … And not like rush and like [real life doctors] can rush, but this [VR doctor] doesn't seem rushing, just talking. (Participant 8)

Participants valued the interactive question‐and‐answer format as it allowed them to direct their learning according to their individual needs and interests. Participant 10 recognised: ‘he gave me a chance to ask some questions. And that's what's most important for me to know.’ This interaction style was appreciated even when participants did not ask any questions during the scenarios, as Participant 9 commented: ‘talking to someone that is… people ask question and then you answer. And then I can say yes or no. Have a choice to say yes or no.’

Some participants recognised the value of the programme for other people, particularly those with limited healthcare experience.

Because I see them before [at my doctor's] and I know what the tools look like. And you know what to do with it. Like first time you see, you see what they do with it. Not everyone know the medical term of the thing. … And I will know this is for the ears, and the thing is for the tongue, for you to look at your tonsils. (Participant 9)

Participants who were familiar with basic healthcare information could identify additional content areas they wanted to explore.

Researcher What would you like [the VR programme] to talk about if you had a choice?

Participant 2 Sexual health… How to make appointments in regards to those sort of things… and things around that section of medical stuff.

Researcher Is that stuff that you get a chance to talk to your doctor about?

Participant 2 Not really. Because I don't know the things to ask or say, about how I'm feeling and what I want to know about. It gets annoying.

The variation in participant responses highlighted the challenge of using standardised content for users with diverse healthcare experiences and information needs. The interactive questioning format, whilst valued by participants, was constrained by the programme's scope in this study, as participants could only ask questions about healthcare items that had already been introduced. This limitation meant that participants seeking information beyond the standardised topics could identify gaps in content relevance but could not access information about their specific areas of interest through the system.

#### 3.2.2 Theme 2: Stepping Into the Virtual Clinic

This theme encompasses participants' experiences of being immersed in virtual healthcare environments and their responses to the realism and authenticity of the VR clinic settings. Participants' experiences varied based on how closely the virtual settings, avatar behaviours, and environmental details matched their expectations and lived experiences. The theme captures both positive experiences of presence and immersion in convincing virtual healthcare spaces and challenges when VR elements felt unrealistic or inconsistent with participants' familiar healthcare environments. All participants contributed experiences related to this theme, noting varying degrees of realism and environmental factors that affected their ability to engage meaningfully with the virtual clinic setting.

When virtual environments aligned with participants' real healthcare experiences, participants described the settings as familiar and realistic. Participant 1 observed: ‘everything is like about the same as how it's set up, like in an actual doctor's room’ and described the waiting room as ‘looks like… actually sitting inside an actual waiting room itself.’ Several participants drew comparisons to mainstream technology experiences, such as Participant 9 likening the experience to ‘seeing movies… 3D movies.’ The similarity to movies or gaming helped align the novel VR experience with technology they were more familiar with and enjoyed using.

When environmental elements contradicted participants' real healthcare experiences, participants noted confusion and raised concerns about realism. Participant 4 found the arrangement of the waiting room and doctor's office disorienting: ‘It's confusing… just the environment… the desk was opposite… This side on the left’ compared to their usual doctor's office. Participant 5 noted practical concerns about equipment sizing: ‘the examination bed, it looked way too small… a full‐size adult needs more room than that.’ Audio‐visual synchronisation problems also affected participants' experiences. Participant 8 noted, ‘Someone [was] coughing, but I didn't even see anyone cough,’ and Participant 10 questioned, ‘where is the cough coming from?’

Some participants described the subjective experience of connection in interactions with the avatars, despite understanding that they were not real. Many participants used politeness markers in their interactions with avatars, such as saying thank you to avatars after getting a response, and at times giving the avatar compliments or asking how they were. Whilst the conversational scope of the avatars was mostly limited to discussing the specific healthcare scenarios presented in the VR programme, they were able to respond appropriately to some of these comments.

Where the avatars' behaviour was consistent with users' real healthcare experiences, this supported their engagement in the scenarios. Participant 10 highlighted the positive impact of the receptionist's behaviour:

This is like you're at the doctor for real. … it's like you're in the real world … it's like someone is really serving you there and asking you a question and being kind to you. … And I feel like I was, I was in my real world. (Participant 10)

This indicates a relationship between participants' sense of presence in the scenario and the relational quality of interactions with avatars. By contrast, when the interactions with avatars lacked positive relational characteristics, this reduced participants' engagement and in one case heightened Participant 10's awareness that he was interacting with AI: ‘But when I was laughing at the doctor, he didn't even notice… It's almost like he's just there to get paid… and is, like, complete AI.’

Many participants compared the AI's communication favourably to their real healthcare experiences, even if there were differences between VR and real healthcare delivery. Participant 8 noted differences between VR and her doctor: ‘they normally sit in the desk to the computer and ask you questions… This one the doctor just talked to you. Like, that is good.’ Participants also highlighted how the AI doctor mirrored real clinical practise by acknowledging limits to their knowledge. Participant 5 said:

He [answered questions] very thoroughly, but he also made sure that anything he couldn't answer he made sure he said go and see, go ask your doctor. … A lot of [doctors] are like that. A lot of them if they don't know they'll go ‘I'm just going to refer you to this specialist to get a more detailed thing’ (Participant 5)

Several participants found the doctor's repetitive hand movements unsettling and interpreted them as signs of nervousness. Participant 3 explained, ‘I didn't like this all the time [mimics doctor's fidget action] … [It made me feel] a bit nervous and wondering what he was thinking,’ and Participant 2 observed the doctor appeared ‘unsure, or nervous… It made me stressed.’ These movements occurred during the consultation scene (Figure 1C), where the doctor avatar remained seated but repeatedly moved his hands whilst speaking. In the waiting room scene (Figure 1B), one background avatar continuously walked back and forth across the room. Whilst Participant 5 viewed this as authentic, Participant 10 found it anxiety‐provoking: ‘one person is just going up and down… that can make someone like people have so much anxiety that can make them have more anxiety.’ These animated behaviours were intended to increase environmental realism; however, participants' responses indicate that dynamic avatar movements can influence emotional comfort in immersive settings.

#### 3.2.3 Theme 3: The Promise and Problem of VR

Participants recognised the potential of AI‐VR technology for learning and healthcare preparation but also identified usability barriers. Technical difficulties disrupted their experience and highlighted current limitations of AI‐based VR systems for people with intellectual disability. Some participants needed help resolving conflicts between the VR headset and assistive devices such as hearing aids and glasses. The AI system's limited conversational capability also emerged as a significant challenge, particularly with the AI doctor avatar. The AI doctor was programmed to respond to questions about healthcare content but did not engage with conversational comments, whereas participants reported positive interactions with the AI receptionist avatar. This difference likely reflects differences in the programming of rules around the avatar interactions. The excerpts shown below reflect the AI‐generated transcript of participants' interactions with the virtual doctor.

AI doctor [Explains how the blood pressure monitor works]. Do you have any questions about blood pressure monitors?

Participant 6 I don't like it when they get, I don't like it. When you put [the blood pressure monitor] on my arm it gets really hard.

AI doctor Now, let's talk about the thermometer.

The AI doctor was programmed to respond to questions about healthcare but had limited ability to respond to comments or statements. Participant 5 captured this difference between AI and human communication: ‘with a human you can talk like that but with a computer… they're very sensitive.’

Speech recognition errors also occurred. Participant 2's experience exemplified these challenges:

Participant 2 Keep going [microphone not green].

Researcher Hold on, just a sec.

Participant 2 Keep going [VR misheard and transcribed as ‘Cape gowing’].

AI doctor It seems like there might be a little mix‐up with the words.

Participant 2 [Interjection] [Expletive].

[Participant took a deep breath, getting frustrated].

AI doctor If you meant ‘keep going,’ I'm here and ready for your next question. If you meant something else, feel free to clarify!

When these issues occurred, the researcher intervened by prompting participants to rephrase statements as questions or by providing guidance when the AI system did not recognise speech accurately. These interventions reduced participants' independent engagement in the scenarios. On some occasions, the researcher prompted participants to rephrase comments as questions. This led to some adaptation in how participants phrased their requests for information, as seen with Participant 2:

AI doctor The otoscope … might tickle a little, but it does not hurt. Do you have any questions about otoscopes?

Participant 2 Sometimes it can hurt, the otoscope.

Researcher (prompts) Have you got a question?

Participant 2 Why does the otoscope sometimes feel uncomfortable?

AI doctor It's possible that if the otoscope feels uncomfortable, it might be due to the position or angle it's being used at… [continues explanation].

In this example, Participant 2 challenged information that did not match their own healthcare experience and requested an explanation. However, researcher intervention was required to help participants rephrase statements as questions when the AI could not respond to comments.

Despite the technical challenges described above, some participants expressed excitement about VR technology. Participant 6 found the AI more responsive than other voice assistants: ‘She never said ‘I don't know’. Not like Siri, who says ‘I don't understand’… those questions [that I ask Siri] are perfectly normal.’ However, this positive experience was not universal, as technical difficulties affected most participants' interactions.

Participants recognised VR's potential as an educational tool that improved upon existing tools such as written information. Participant 9 noted: ‘I think it's the technology now… you can see like movie… Having more fun… To see those pictures inside, rather than just, you know, ask question and a list of paper.’ Participants also saw VR as a way to enhance telehealth, for example by enabling virtual healthcare appointments. Participant 1 explained: ‘if, like, you can't make it to get out to your door to go to your doctor. [You could have a] virtual reality appointment saying, look, we can get your medication sorted.’

Some participants viewed the VR programme as a way to train health professionals to communicate more effectively with people with intellectual disability, highlighting its potential to shape more positive perceptions of healthcare. Participant 3 explained:

… it's a good way of getting information. … A lot of people wouldn't know how to go [to] the doctor and like, by you doing this probably will help train other doctors to …[meet] the disability person's [needs]. … Or it makes [people] think [the doctor's] is a lot better than what it is. A lot of people might not want to go to the doctor. (Participant 3)

Participants suggested ways the VR programme could be expanded or improved. Participant 7 suggested adding a virtual support person in the waiting room scenario who could provide companionship and reassurance to help reduce anxiety for people with intellectual disability. ‘It is good to be able to help, help ones with disability, … this needs to have someone to actually talk to in the waiting [room] … Just to assure them… it's not scary, that it's not scary or something’ (Participant 7).

Participants showed adaptation in their communication in interactions with the AI avatars. The AI system occasionally provided helpful communication support, as illustrated by this interaction between Participant 5 and the AI ‘receptionist’:

AI receptionist …. Do you have any questions about concession cards?

Participant 5 Oh, No. Actually, I don't know how to say it actually. Um.

AI receptionist That's alright, take your time. If you have any questions or need clarification about concession cards, feel free to ask when you're ready.

Researcher (prompts) Do you have a question you want to ask?

Participant 5 Can you use your concession card and Healthcare card? Or can you only use one of them?

AI receptionist You can have both a concession card and a Health Care Card… [continues explanation].

## 4 Discussion

This study co‐designed, developed, and evaluated the feasibility and usability of an AI‐based VR application for healthcare preparation in people with intellectual disability. All participants completed the VR tasks and engaged in dialogue with AI avatars, demonstrating that the technology was accessible and acceptable to this population. Participants valued the AI communication style, which was perceived as patient and unrushed. This is notable given that several participants reported at baseline that they only sometimes understood what doctors told them and did not always feel listened to, suggesting that the structured and clear communication within VR may have addressed gaps in their usual healthcare experiences. The interactive format allowed participants to practise asking questions, learn medical terminology, and familiarise themselves with clinical environments and procedures. However, speech recognition errors and microphone activation timing presented challenges for some participants, and the AI's limited ability to respond primarily to questions rather than conversational comments constrained natural dialogue in some instances.

The AI system's conversational scope helped guide users but also restricted participants' ability to engage in natural dialogue, particularly with the AI doctor avatar. The guided and structured dialogue helped participants know when to speak and what to expect next (Mateos‐Sanchez et al. 2022). However, participants could not always engage in the type of natural conversational exchange they might expect in real healthcare interactions, where building rapport through small talk, casual conversation, and responsive communication is essential for establishing trust and comfort (Mimmo et al. 2022; Nijhof et al. 2024; Chandra et al. 2022). The AI‐doctor avatar was programmed to respond primarily to questions about specific healthcare content, causing difficulties when participants attempted casual conversation. In contrast, the AI receptionist demonstrated greater conversational flexibility, indicating that the doctor's constraints reflected design choices about providing healthcare information rather than technological limitations. These relational cues are particularly important for people with intellectual disability, for whom structured but emotionally responsive communication supports engagement and learning (Mateos‐Sanchez et al. 2022; Huq et al. 2024).

Technical usability shaped how participants engaged with the AI‐VR system, revealing both strengths and areas for improvement. The visual design and scene transitions supported focus and orientation, and most participants were able to converse naturally once the AI avatars recognised their speech. The headset was comfortable for most users, and the clear visual cues helped participants follow the conversation flow. Participants valued the avatars' patient pacing, noting that short pauses between responses gave them time to think and reduced pressure to respond quickly. However, technical issues occasionally interrupted this flow. Speech recognition errors and microphone activation delays sometimes prevented the AI from registering spoken input, leading to frustration and loss of conversational rhythm in some instances. Some participants needed help adjusting the headset or progressing to the next scene, which reduced independent engagement. Overall, participants demonstrated that with minor support, people with intellectual disability can engage meaningfully with AI‐based VR, but refinements to interaction design and hardware usability are needed to enable full autonomy.

The study revealed tensions between creating realistic healthcare environments and maintaining accessibility for people with intellectual disability. Features designed to enhance immersion, such as ambient noise or moving avatars, increased anxiety for some participants whilst others found them authentic. This variation aligns with baseline reports that many participants felt anxious or nervous about attending medical appointments, indicating that immersive realism may interact with pre‐existing emotional experiences of healthcare. This challenges traditional VR design approaches that prioritise presence and immersion through environmental realism (Wilkinson et al. 2021), suggesting these features may generate stress for some people with intellectual disability (Franze et al. 2024; Langener et al. 2022). These findings raise important questions about the goals of VR healthcare preparation. One approach would prioritise comfort by removing anxiety‐provoking elements. However, this may limit opportunities to practise strategies for managing realistic but uncomfortable healthcare situations, such as busy waiting rooms. An alternative approach could offer adaptable environments that allow gradual exposure to challenging sensory features as users develop coping strategies, if that aligns with their goals. This could support preparation for healthcare settings where quieter spaces or other reasonable adjustments may not be available. The customisable nature of VR technology makes it particularly suited to accommodating individual preferences and needs, allowing users to practise in environments that match their actual healthcare settings or to develop tolerance for challenging features over time.

This study has methodological limitations that affect interpretation and generalisability of findings. All participants were recruited through a disability advocacy organisation, suggesting they were experienced advocates compared to the broader population of people with intellectual disability. All participants were currently engaged with healthcare services, with access to a consistent general practitioner or medical practise when needed. This limits insights into barriers faced by those who avoid healthcare entirely or have less confident self‐advocating. Our inclusion criteria, which required verbal communication and safe use of VR equipment, along with exclusion of people with epilepsy based on VR safety guidance, limit the applicability of findings to people with intellectual disability who use alternative communication methods or cannot use head‐mounted displays. The study involved only a single exposure to the VR system, which may not reflect learning that occurs with repeated use or assess retention of skills over time. The researcher equipped participants with the VR headset and retained control of the handheld controller throughout sessions to improve efficiency, but this may have limited assessments of usability. The study relied on self‐report data without objective measures of learning or confidence changes, and the laboratory setting may not reflect how the technology would perform in real community environments.

This study demonstrated that AI‐based VR applications have potential for healthcare preparation in people with intellectual disability, though significant usability barriers currently limit independent use. Participants valued the patient communication style of AI avatars, but speech recognition difficulties and the AI's inability to respond to conversational comments required frequent researcher intervention. The findings highlight that effective VR design must balance environmental realism with user comfort, as immersive features can increase anxiety. Future development should prioritise AI systems capable of natural dialogue and speech recognition that accommodates diverse communication patterns. Co‐design with people with intellectual disability remains essential for developing accessible AI‐VR healthcare applications.

## Author Contributions

Funding was secured by S.C.M. (lead Chief Investigator) with J.N.T., S.M.G., A.D., and R.C.C. (Chief Investigators). The study was designed by S.C.M. with input from A.D., R.C.C., S.M.G., and J.N.T. Recruitment and data collection were completed by J.A. Thematic analysis was completed by S.C.M. and J.A. This paper was written by S.C.M. with further critical revision and final approval by all authors.

## Funding

This research was funded by the UNSW Disability Innovation Institute Research Seed Funding 2024 and the UNSW Health Systems Research Theme 2025 Collaborative Grant. R.C.C. was supported by a Dementia Australia Research Foundation funded by the Dementia Australia Research Foundation and Bartle Pathway to Care. J.N.T. receives funding from several sources including the following National Health and Medical Research Council Australia grants: Understanding health service system needs for people with intellectual disability (GNT1123033) and Addressing health inequality experienced by people with intellectual disability (GNT2009771). The National Centre of Excellence in Intellectual Disability Health is funded by the Australian Government Department of Health, Disability and Aged Care.

## Ethics Statement

This study was granted ethics approval from the UNSW Sydney Human Research Ethics Committee (HREC6912).

## Conflicts of Interest

The authors declare no conflicts of interest.

## Data Availability

The data that support the findings of this study are available on request from the corresponding author. The data are not publicly available due to privacy or ethical restrictions.
